# High-Throughput Microbore LC-MS Lipidomics to Investigate APOE Phenotypes

**DOI:** 10.1021/acs.analchem.3c02652

**Published:** 2023-12-19

**Authors:** Darshak Gadara, Vratislav Berka, Zdenek Spacil

**Affiliations:** † RECETOX Centre, Faculty of Science, Masaryk University, Brno 625 00, Czech Republic

## Abstract

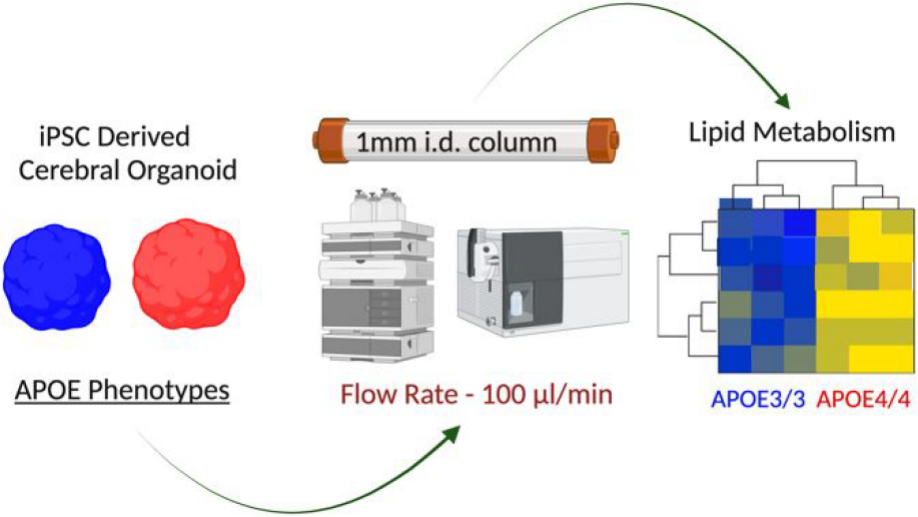

Microflow liquid chromatography interfaced with mass spectrometry (μLC-MS/MS) is increasingly applied for high-throughput profiling of biological samples and has been proven to have an acceptable trade-off between sensitivity and reproducibility. However, lipidomics applications are scarce. We optimized a μLC-MS/MS system utilizing a 1 mm inner diameter × 100 mm column coupled to a triple quadrupole mass spectrometer to establish a sensitive, high-throughput, and robust single-shot lipidomics workflow. Compared to conventional lipidomics methods, we achieve a ∼4-fold increase in response, facilitating quantification of 351 lipid species from a single iPSC-derived cerebral organoid during a 15 min LC-MS analysis. Consecutively, we injected 303 samples over ∼75 h to prove the robustness and reproducibility of the microflow separation. As a proof of concept, μLC-MS/MS analysis of Alzheimer’s disease patient-derived iPSC cerebral organoid reveals differential lipid metabolism depending on APOE phenotype (E3/3 vs E4/4). Microflow separation proves to be an environmentally friendly and cost-effective method as it reduces the consumption of harmful solvents. Also, the data demonstrate robust, in-depth, high-throughput performance to enable routine clinical or biomedical applications.

Current lipidomics studies aim to profile hundreds of lipid species from biological specimens to investigate lipid homeostasis.^1^ Among other techniques, standard flow liquid chromatography (250–600 μL/mL) interfaced with mass spectrometry (MS) remains dominant in pursuing lipidomics analyses.^2,3^ Research laboratories are increasingly moving toward high-throughput 3D in vitro assays, and individual samples are subjected to multiomics or broad-spectrum molecular species monitoring to understand molecular mechanisms.^4^ Miniaturization of the instrument has become the leading trend as nanoliquid chromatography coupled to MS has proven to be a workhorse in proteomics^5^ and was applied to improve in-depth lipid characterization^6−9^ in small-volume biological samples. However, reproducibility, long run times, and maintenance challenges hamper the broader penetration of nanoflow into metabolomics and lipidomics studies.^10^

On the other hand, microflow chromatography (<100 μL/mL) enables a balance between sensitivity and reproducibility, as recently demonstrated in various proteomics studies.^11−14^ Microflow separation is enabled in a standard UHPLC system using 1 mm i.d. columns, and was successfully evaluated and implemented in proteomics^12,13^ and metabolomics^15,16^ studies. Previous studies have employed microbore separation to pursue lipidomics.^17−20^ However, a study collectively presenting microbore separation method optimization is needed as a reproducibility assessment, with benchmarking against a standard narrow-bore column.

Cerebral organoids (COs) derived from pluripotent stem cells recapitulate the human brain’s complexity and are a functional alternative to exploring the etiology of neurological disorders, including Alzheimer’s and Parkinson’s diseases.^21,22^ Omics technologies applied to an iPSC-derived cell biology model system are a powerful approach to investigating phenotypes and underlying disease pathophysiology.^23−25^ Here, we have focused on developing single CO lipidomics to achieve high analytical throughput and avoid commonly used pooling of biological replicates that are tedious, costly, and compromise statistical evaluation.

We evaluated the microbore column in an exploratory lipidomics study using a standard widely available UHPLC system. We use a 1 × 100 mm RPLC column at a 100 μL/min flow rate coupled to a three-stage quadrupole mass spectrometer. The optimized reversed-phase gradient condition achieved a ∼4-fold increase in response compared to that of conventional lipidomics methods. Further, we probed the lipid composition of a single iPSC cerebral organoid, and more than 300 samples were acquired over 75 h to prove the quantitative performance. We have shown that microflow separation allows cholesterol measurement in a single lipidomics method, simultaneously with other lipid classes. As a proof of concept, we studied the difference in lipid metabolism of APOE phenotypes of iPSC cerebral organoids. Our study reveals that APOE4/4 causes widespread lipid dysregulation in a relevant disease model.

## Experimental Section

Detailed information on chemicals, reagents, and cell culture is provided in the Supporting Information.

### Sample Preparation

Cerebral organoids (COs) were washed with PBS, treated for 1 h with cell recovery solution (CRS, Corning, NY, USA) at 4 °C, and rewashed with PBS. For targeted lipidomics analysis, a single CO was used; ten biological replicates per condition were acquired in technical duplicates. CO was homogenized in a microtube with a glass bead (Benchmark Scientific, Edison, NJ, USA). A 100 μL portion of 80% IPA was added to the homogenate for lipid extraction. The sample was vortexed (1 min), sonicated (37 Hz, 5 min), and mixed (10 min, 2000 rpm). Then 85 μL of the supernatant was removed and mixed 1:1 with a mixture of internal standards (Table S2) for lipidomics and stored at −20 °C until LC-MS analysis. The sample was injected in 80% IPA. The removed protein pellet was dried in the SpeedVac vacuum concentrator (Savant SDP121 P, ThermoFisher Scientific). The dried protein pellet was consequently reconstituted to perform the BCA protein determination.

### Experimental Conditions of μLC-MS/MS

The lipid extract was analyzed using 1290 Infinity II UHPLC (Agilent) with a low void volume mixer (10 μL) coupled to 6495 Triple Quadrupole mass spectrometer (Agilent). The lipid extract (1 μL) was injected on the reverse phase microbore column (CSH, 1 × 100 mm, 1.7 μm, Waters) and separated in gradient elution mode at 100 μL/min flow rate over 15 min. The mobile phase A was 10 mM ammonium formate in acetonitrile/water (60:40), and the mobile phase B was 10 mM ammonium formate in isopropanol/acetonitrile (90:10). A 1 mL portion of Milli-Q water was used to dissolve ammonium formate, which was then added to the mobile phase B bottle. The mixture was shaken vigorously and sonicated for 5 min to ensure solubility. The gradient elution program was as follows: 0 min 15% B, 1.86 min 30% B, 2.32 min 48%, 9.5 min 82% B, 12.5 to 13.5 min 99% B, and 13.5 to 15 min column re-equilibration. The positive ion mode parameters of the jet stream were: gas temp 200 °C, gas flow 14 l/min, nebulizer pressure 45 psi, sheath gas temp 400 °C, and sheath gas flow 8 l/min; capillary voltage 4 kV, nozzle voltage 500 V, and unit resolution for Q1 and Q3. Data were acquired in positive ion mode using the dynamic selected reaction monitoring (SRM), with a 2 min retention time window per transition.

Batch samples were analyzed on the same day in a randomized order to minimize internal variation. After every four-matrix injection, a blank sample was submitted to avoid carryover and ensure chromatography column and ion source cleaning. QC samples were injected after every eight samples throughout the batch analysis to monitor reproducibility by calculating the coefficient of variation (%CV). Only lipid species with CV < 30% were considered for biological interpretation.^26^

### Data Processing and Statistical Analysis

Raw data files were processed using Mass Hunter Quantitative analysis (B.07.00, Agilent Technologies) software. Lipids were quantified using the SRM transition reported in the literature.^27−29^ Collision energies were optimized by directly injecting a mixture of 16 class-specific internal standards. Lipid nomenclature was followed per the LIPID MAPs consortium guidelines.^30^ Identified lipid species were separated based on retention behavior of carbon number or double bond of homologous lipid series.^31^ Only the sum composition of lipid species was provided as high-resolution MS/MS was not acquired for the more profound structure elucidation. The study aimed to conduct a case/control comparison; therefore, only relative concentrations of lipid species were calculated using single-class-specific internal standards. However, the literature presents alternative methods for reporting absolute lipid concentration, which may be particularly useful in clinical studies or studies where the data must match with global or in-house reference values.^32^ The relative lipid concentrations were further normalized to the protein content to account for the variability in organoid cellular mass. The average protein concentration for an individual cerebral organoid was ∼31 μg.

## Results and Discussion

### Optimization of Gradient and Flow Rate for the Microbore Column

**Figure 1:**
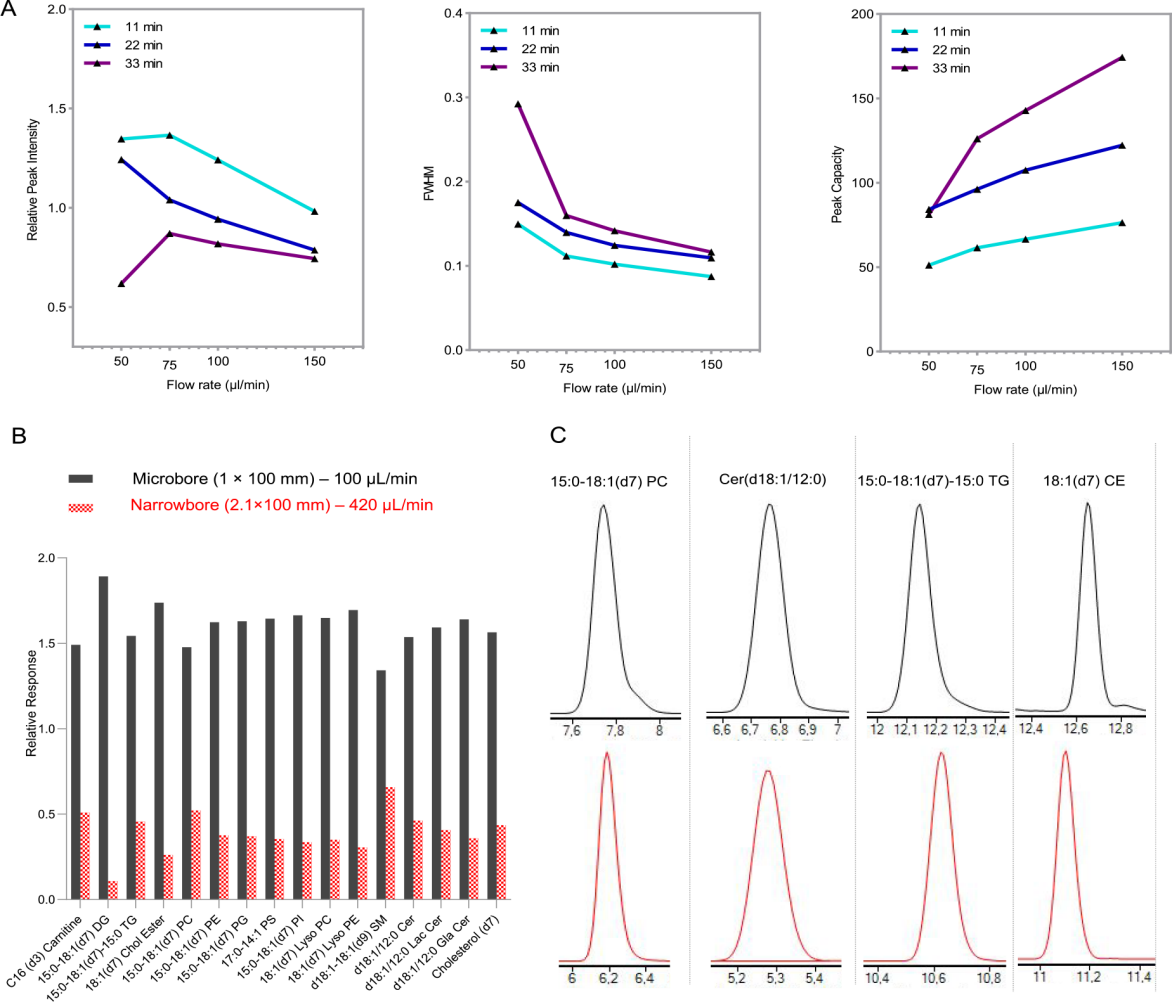
(A) Optimization of lipidomic gradient conditions using a microbore column (1 × 100 mm). Fifteen lipid standards were analyzed at 12 gradient conditions, including four different flow rates (50, 75, 100, and 150 μl/min) with three different gradient durations (11, 22, and 33 min). Summary of the relative average peak intensity, full width at half maximum (FWHM), and average peak capacity. (B) Comparison of microbore (1 × 100 mm) and conventional narrow bore column (2.1 × 100 mm), depicted in black and red, respectively. Microbore separation increases the response 3.7-fold at a comparable peak width.

A robust LC-MS instrument is capable of analyzing many samples while maintaining the analytical performance. LC parameters require careful optimization, as they influence the number of identifications, ion suppression, and the overall experiment reproducibility. First, we investigated the performance of a microbore (1 mm i.d.) column under 12 linear gradient conditions (Table S3), including various flow rates (50, 75, 100, and 150 μL/min) and linear gradient durations (11, 22, and 33 min). A mixture of 15 lipid standards (Table S2) was analyzed to assess the class-specific influence on the LC conditions. UHPLC hardware modifications to reduce extra column band broadening associated with microbore columns were reported.^12,13^ We replaced column outlet tubing with 50 μM i.d. nanoViper (Thermo). Figure 1A summarizes the average peak intensity, full width at half maximum (FWHM), and peak capacity for the tested gradient conditions.

As expected, the average peak intensity and FWHM decreased as the LC flow increased. A peak broadening during longer gradients decreased the average peak intensity. Our data suggest that a 50 μL/min flow rate combined with a 33 min gradient results in broad peaks and a dramatic decrease in peak intensity. The peak capacity for each tested flow rate increased with the gradient length, as shown in Figure 1A. However, this is at the expense of a reduced peak height and increased instrument time. Consequently, a reduced signal-to-noise ratio hampers the detection of the low-abundance lipid species. We choose the 11 min gradient duration for the final method as it resulted in maximal peak intensity with the narrowest peak width. Reduced flow rate improves the ionization efficiency and hence increases peak intensity, but causes peak broadening. Also, low flow rates (50 and 75 μL/min) did not elute the hydrophobic lipid species, such as CE and TG at short gradient duration. Therefore, we chose a 100 μL/min flow rate and 11 min gradient duration for the lipidomics sample analysis. The total gradient duration was 15 min including an additional 2.5 min nonpolar solvent elution of longer chain TG and CE, followed by 1.5 min equilibration time.

To benchmark the microbore column (1 × 100 i.d. mm) performance with the high-flow narrow bore column (2.1 × 100 i.d. mm), we employed optimized gradient conditions (flow rate 100 μL/min, 15 min gradient length). The narrow bore column flow was 420 μL/min to keep the constant linear velocity. We analyzed 15 lipid internal standards (Table S2) to assess the difference in peak intensity and FWHM. As lower LC flow rates compromise reproducibility, our study focused on optimizing gradient conditions and probing the quantitative performance of batch analysis.

Interestingly, we observed a significant increase in peak intensity (Figure 1B) for the DG (16-fold) and CE (6.8-fold). A reduced flow rate probably improves the ionization efficiency of the relatively hydrophobic lipid species. For microbore separation, the FWHM of all lipid standards was 0.08–0.22, similar to the peak width on narrow bore elution (Figure 1C), except for early eluting lipids such as LPC, LPE, and carnitines, which produced broader peaks in microbore separation. The peak intensity and FWHM for 15 lipid internal standards for the microbore and narrow-bore separation are provided in Table S4 and Table S5. In-depth structural annotation could not be pursued during the triple quadrupole analysis. However, our data demonstrated a similar peak resolution compared to narrowbore separation for the isobar and isomer. Figure S1 depicts the relative elution profile of the SRM transition (746.5 > 184), which represents the three phospholipid species (PC 33:1, PC O-34:1, and PC P-34:0). Figure S2 shows the relative separation of the four different species of triglycerides (TG 52:0, TG 52:1, TG 52:2, and TG 52:3).

### Application to Single iPSC-Derived Cerebral Organoid (CO) Lipid Profiling and Batch Analysis of 303 Samples

**Figure 2:**
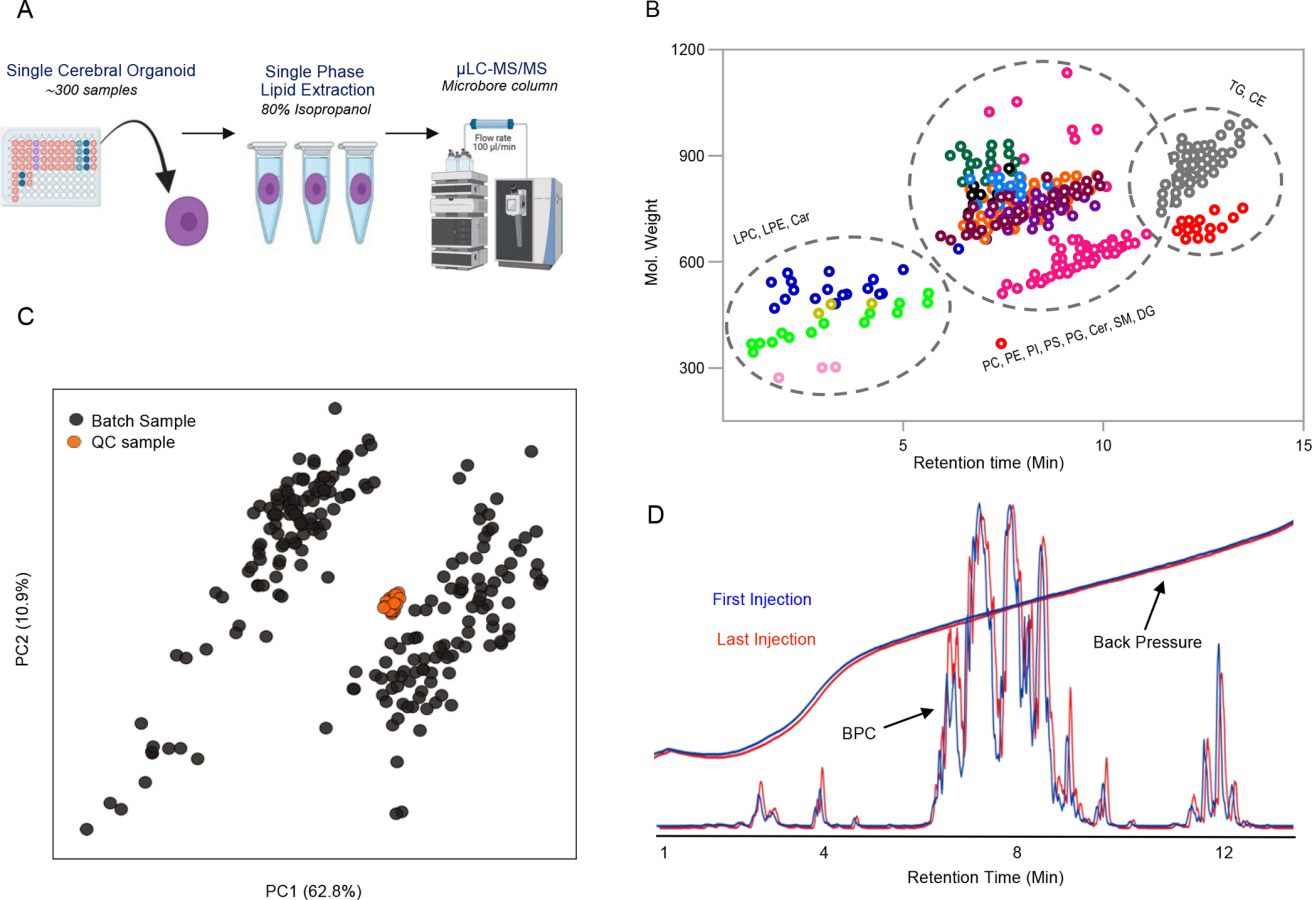
Single iPSC Cerebral Organoid (CO) lipidomics using μLC-MS/MS. (A) Each CO sample was subjected to single-step lipid extraction (80% isopropanol) and injected for a lipidomics profiling (15 min). (B) Relative elution of 351 lipid species quantified from a single CO. (C) Consecutive 303 injections acquired for the assessment of reproducibility. PCA plot shows a tight clustering of pool QC samples injected after every eight injections during the batch analysis. (D) Overlay of the base peak chromatogram and back pressure of the first (green) and last (black) injection demonstrate the high stability of microbore lipid separation during the batch analysis.

Our optimized microflow LC-MS/MS method (positive ion mode, 15 min gradient duration) identified 351 lipid species across 23 lipid classes from the lipid extract of a single cerebral organoid. The average size of a single cerebral organoid is equivalent to ∼40 μg protein. Generally, biological assays require a pool of organoids or spheroids, which is technically challenging and costly. A microscale separation was interfaced with a three-stage quadrupole mass spectrometer to substantially boost the sensitivity to leverage high-throughput single cerebral organoid lipidomics (Figure 2A). We profiled iPSC-derived cerebral organoids using a dynamic SRM method to routinely probe the lipid metabolism associated with a disease or a perturbation. Relative retention in reversed-phase microbore column of lipid classes LPC, LPE, SM, Cer, HexCer, PC, PE, PI, PG, PS, DG, TG, Cholesterol, and CE is depicted in Figure 2B. Certain anionic lipid classes (CL, BMP, PA, and PIP) and free fatty acids (FA) were excluded from the analysis as they preferentially ionize in negative ion mode.^33^ If not baseline separated, BMP 36:2 can contribute to quantitative results of the structural isomer PG 36:2 as they share the same SRM transition in positive ion mode. Polarity switching is proposed to sensitively analyze the compound of diverse physiochemical polarity into a single LC-MS run.^34,35^ The pseudo-SRM is used for the lipid classes such as fatty acids, which poorly fragment into electrospray ionization.^36^

Lipidomics method reproducibility is essential for biological interpretation. Quantitative performance depends on peak shape, stable instrument response, retention time, etc. The 2.1 mm i.d. narrow bore column has been a workhorse for lipidomics studies; robustness is widely accepted. We demonstrate the reproducibility of a 1 mm i.d. microbore column, continually analyzing lipid extract from 220 single cerebral organoid samples spiked with 16 lipid internal standards for the single point calibration of 351 lipid species. A QC sample was injected after every eight injections to monitor reproducibility, and a blank sample was injected after every four injections to clean the column. In total, 303 injections were continually acquired over 75 h. Principal component analysis (Figure 2C) shows a tightly clustered pool QC sample, which shows the reproducibility of a method during the batch analysis. Two separate groups on the PCA plot represent the APOE phenotypes 3/3 and 4/4. Figure 2D depicts the superimposed base peak chromatogram and LC system backpressure trace between the first and last injection of batch analysis. A percentage coefficient of variation (%CV) < 30 is acceptable in narrow bore separation of lipids species.^27,37−39^ In the present study, the median coefficient of variation (CV) of 351 lipid species was 12.95% and only 26 showed a CV >30%. The median coefficient of retention time variation was 0.1%, indicating stable and robust LC gradient elution conditions. Our data demonstrate the robustness of microbore columns to be applied to large-scale lipidomics studies. While we expect that the increased sensitivity will also benefit untargeted lipidomics, the potential of microbore versus narrow bore separation for such analyses still needs to be evaluated.

### Reproducible Measurement of Cholesterol by Microbore Separation

**Figure 3:**
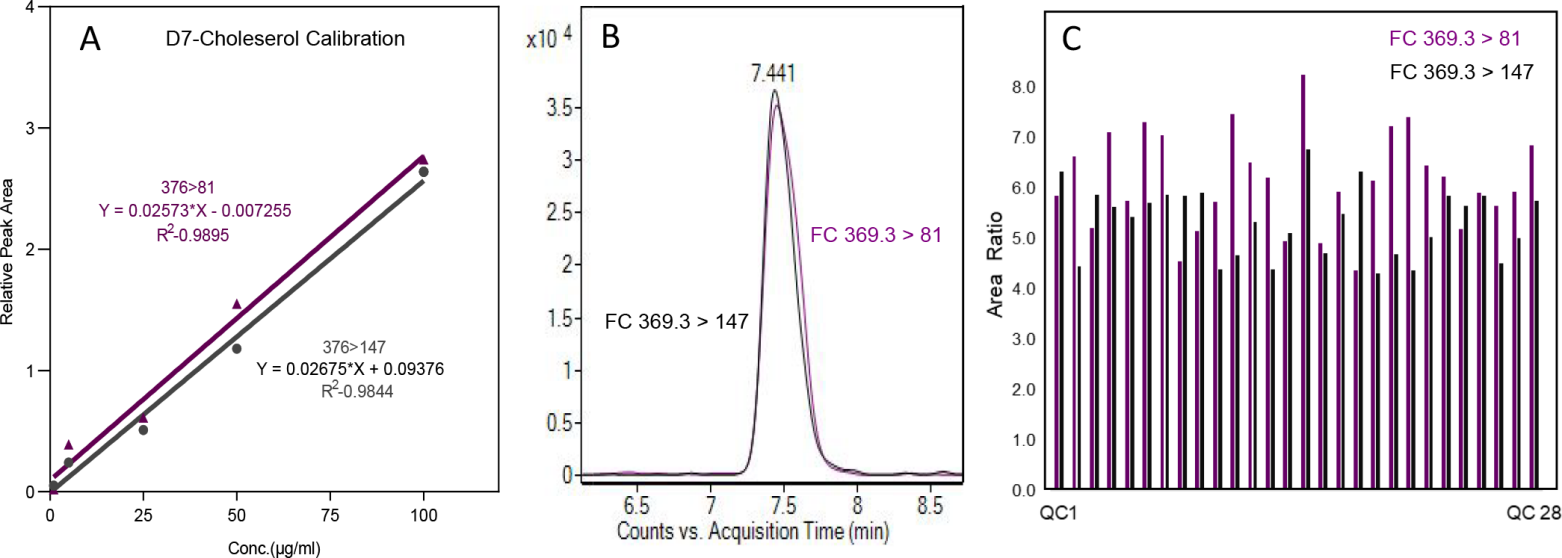
Sensitive and reproducible measurement of cholesterol using microflow LC-MS/MS. (A) Standard curve and goodness of fit (*R* square) for d7-cholesterol spiked in cerebral organoid matrix, SRM quantifier 376 > 81 and qualifier 376 > 147 transitions. (B) LC-MS response of cholesterol from single cerebral organoid lipid extract. (C) Reproducibility of cholesterol measurement from the pool QC sample injected throughout batch analysis of 303 samples.

Free cholesterol (FC) maintains the cell membrane fluidity and lipid homeostasis. Therefore, measuring free cholesterol and cholesteryl ester (CE) is paramount in clinical and basic science.^40^ Electrospray ionization mass spectrometry (ESI-MS/MS) is applied routinely to quantify CEs and other lipids classes. Poor ionization of FC in ESI impedes the quantification using UHPLC-MS/MS. Therefore, most lipidomics studies measured cholesterol independently using acetate^40^ or sulfate^41^ derivatization to improve electrospray ionization or other techniques such as GC-MS, APCI-MS, etc.,^42^ which is cumbersome and time-consuming. A recent article reveals that cholesterol measurement is not included in LC-MS lipidomics of cerebral organoid samples.^43,44^ In principle, the ESI ionization efficiency improves with low flow rates. Our microscale method (100 μL/min) resulted in a ∼4-fold increase in response to all lipid standards compared to the standard separation method (420 μL/min). It compelled us to test the electrospray ionization of free cholesterol by using microscale separation. SRM transitions were optimized for d7-cholesterol by injecting (LC flow of 50 μL/min) directly into a three-stage quadruple mass spectrometer. ESI MS/MS spectra are depicted in Figure S3. Two best-performing transitions were selected: quantifier (376 > 81) and qualifier (376 > 147). Furthermore, d7-cholesterol was spiked into cerebral organoid lipid extract in the 1–100 μg/mL dilution series (Figure 3A), resulting in R2 > 0.9 linear regression range for both transitions. We measured cholesterol and other lipid species in a single lipidomics method in a batch analysis of 303 samples as explained in the previous section. Assessment of the QC sample reveals excellent reproducibility, indicating a coefficient of variation of 18.79% for the quantifier and 14.10% for the qualifier (Figure 3C). Application of our method reveals perturbation of cholesterol metabolism in APOE phenotypes, which is discussed in a subsequent section. In summary, we have shown that microbore separation coupled with QqQ mass spec allows the sensitive and robust measurement of cholesterol and other lipid classes in a single lipidomics method, significantly increasing the throughput of sample analysis.

### APOE Phenotypes Reveal Distinct Lipid Profile

**Figure 4:**
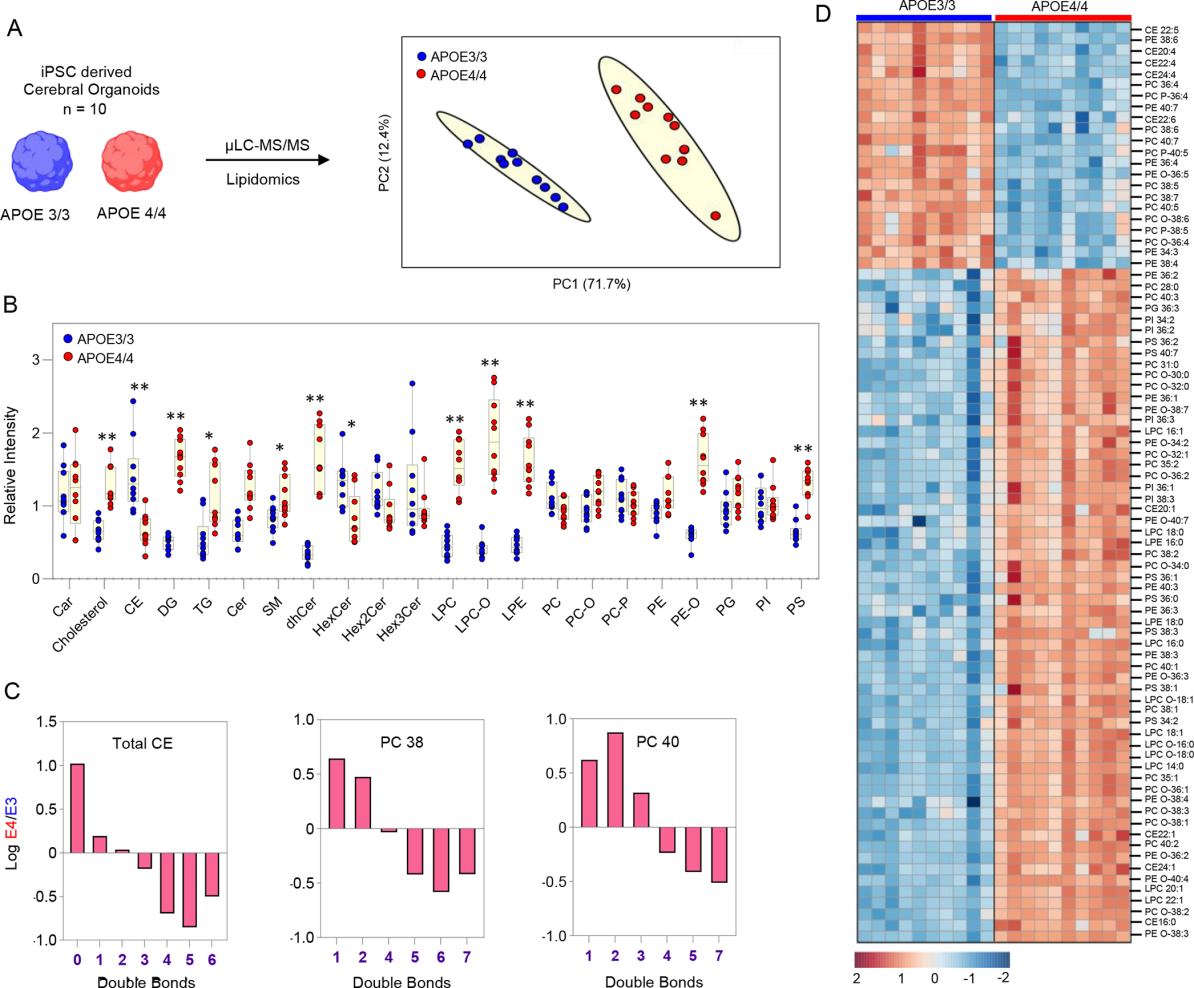
APOE phenotypes (E3 and E4) show differential lipid metabolism in (A) principal component analysis of APOE 3/3 and APOE 4/4 iPSC AD cerebral organoid (*n* = 10). (B) Relative response of major lipid class between APOE phenotypes. (C) Lipid species with 4 or more double bonds are downregulated in E4 CO, shown for total CE and two species of phosphatidylcholine (PC 40 and PC 38); the emaining individual species shown as heat map (D). Mann–Whitney test performed using GraphPad Prism (9.3.1); * < 0.05, ** < 0.01.

The E4/4 allele of the Apolipoprotein (APOE) gene is considered an important genetic risk factor for Alzheimer’s disease. However, the underlying pathophysiological mechanism is not clear.^45^ Apolipoprotein plays a crucial role in the brain by transporting cholesterol to maintain cell membrane homeostasis. APOE4 is shown to be hypo-lipidated compared to APOE3, and pathological effects are linked to the disruption of lipid metabolism.^46^ Therefore, we performed global lipidomics to elucidate the difference in lipid metabolism between the APOE3/3 and APOE4/4 phenotypes of AD patient-derived iPSC cerebral organoids. Lipid extract of APOE3/3 and APOE4/4 (*n* = 10 biological replicates per condition) cerebral organoids were subjected to analysis using the optimized microflow LC-MS/MS method. Principal component analysis (Figure 4A) of 351 lipid species (23 lipid classes) shows a significant change in the lipid metabolism of the APOE phenotypes.

Next, we investigated APOE phenotype-dependent change in the lipidome at the level of lipid classes (Figure 4B) and species. Most of the glycerolipids (GPs), such as triglycerides (TGs) and diglycerides (DGs), were accumulated in the E4/4 cerebral organoid. Several publications have observed E4-dependent lipid droplet accumulation or hypertrigyecerenia.^47^ We see upregulation of cholesterol level on the E4/4 phenotype, in contrast to downregulation of downstream metabolite cholesteryl ester. In mammals, cholesterol synthesizes steroidal hormones and is required for cell membrane and lipid draft formation. Cholesterol dysregulation poses a profound risk to the normal function of the brain. Therefore, the relationship between altered cholesterol metabolism and neurodegenerative disorder is actively investigated using various in vitro and preclinical model systems.^45^ A study on iPSC-based brain cells revealed that APOE4 decreases the astrocyte’s apolipoprotein level and increases the level of cholesterol.^48^ Cholesteryl ester significantly improves cholesterol transport, as it can be packed into the interior of lipoprotein particles. Downregulation of cholesteryl ester indicates compromised cholesterol transport in E4 phenotypes,^49^ potentially linked to the pathophysiology of Alzheimer’s disease. An in-depth phospholipid analysis shows no significant change in total phosphatidylcholine, phosphatidylethanolamine, phosphatidylinositol, and phosphatidylglycerol levels between APOE phenotypes, except for the upregulation of total PS, PE O, and lysolipids LPC and LPE in APOE4/4 cerebral organoids. Interestingly, most lipid species with 4–7 double bonds from the PC, PE, and PI were downregulated in the APOE4/4 phenotype, while lipid species with 0–3 double bonds showing no change or upregulation. Figure 4D depicts the heatmap showing the relative intensity of lipid species (*p* < 0.05, 2 ≥ fold change) from the CE and phospholipids. Regardless of the unsaturation status of acyl chains, all the lipid species of PS were upregulated in APOE4/4. Saturated fatty acids increase membrane rigidity. Oppositely, unsaturated fatty acids like PUFA enhance membrane fluidity, thus controlling various biological functions.^50^ The study reveals that only fatty acids containing 4 or more double bonds enhance cell membrane fluidity and APP. Fatty acids with 3 or fewer double bonds show no effect.^51^ Therefore, our data suggest that APOE4/4 impaired the esterification of polyunsaturated fatty acids (4 or more double bonds) into phospholipids and cholesteryl ester. APOE4/4 shows increased ceramides, sphingomyelins, and dihydroceramides while decreasing hexosylceramides. Gene expression study has shown a gradual increase in ceramide during the decline of cognitive function and a decrease in the synthesis of glucosyl ceramide.^52^ Our study demonstrated widespread lipid dysregulation in the APOE4/4 iPSC cerebral organoid system for the first time.

## Conclusion

We demonstrated the application of 1 mm inner diameter microbore column-based separation to perform exploratory lipidomics analysis. The gradient conditions optimized microbore separation using a standard UHPLC system to achieve the optimal performance. The developed method shows an average 3.7-fold increase in response compared with the conventional lipidomics method while maintaining a similar peak width. The presented single-shot μLC-MS/MS method quantifies 351 lipid species from 23 lipid classes from the single iPSC-derived cerebral organoid in 15 min. Consecutively, we analyzed 303 samples over 75 h to benchmark the quantitative performance. The median coefficient of variation (CV) of 351 lipid species was 12.95%, and only 26 lipid species showed a CV higher than 30%. We have shown that microflow separation coupled to three-stage quadruple MS substantially increases the sensitivity and allows robust cholesterol measurement in a single method with other lipid species from a single cerebral organoid sample. Here, we deliberately processed a single cerebral organoid to enable a high-throughput in vitro assay and sample preparation and analyzed the samples only in positive ion mode to achieve an analytical throughput.

Finally, the optimized high-throughput lipidomics method was applied to elucidate the difference in lipid homeostasis between APOE3/3 and APOE4/4 AD iPSC cerebral organoids. Our results reveal that APOE4 causes widespread lipid dysregulation, including cholesterol and sphingolipid metabolism. Interestingly, our data reveal that the APOE4/4 phenotype interferes with the incorporation of polyunsaturated fatty acids (4 or more double bonds) into phospholipids and cholesteryl esters. Thus, we have revealed APOE4/4-induced lipid alteration in a human-relevant 3D model, which could potentially link to the pathophysiology of Alzheimer’s disease (AD). Further, the developed workflow extended to elucidate the phenotype-dependent lipid dysregulation of tramiprosate,^53^ a pharmaceutical candidate developed to treat AD patients.^54^ Our report is the first to demonstrate that a microbore column-based LC-MS platform enables sensitive, high-throughput, and robust measurement of the global lipidome. This work paves the way for more applications of microflow separation in lipidomics.

## Supporting Information Available

Chemicals and reagents; cerebral organoid culture; characterized lipid species of single cerebral organoid (Table S1); concentration (Table S2); gradient and flow rate optimization for the microbore column (Table S3); peak height of lipid internal standards for microbore and narrow-bore separation (Table S4); fwhm of 16 lipid internal standards for microbore and narrow-bore separation (Table S5); relative chromatographic separation for three phospholipid species PC 33:1, PC O-34:1, and PC P-34:0 (Figure S1); relative chromatographic separation for four triglyceride species TG 52:0, TG 52:1, TG 52:2, and TG 52:3 (Figure S2); ESI MS/MS spectra of d7-cholesterol (Figure S3); Lipidomics Standards Initiative reporting checklist (PDF)
