# Supplementary material for: High-Throughput Microbore LC-MS Lipidomics to Investigate APOE Phenotypes
Source: Anal Chem. 2023 Dec 19;96(1):59–66. doi: 10.1021/acs.analchem.3c02652 (PMC10782415; doi:10.1021/acs.analchem.3c02652)
Supplement: Supplementary file 1 — ac3c02652_si_002.pdf [file ac3c02652_si_002.pdf]

Supporting Information

High-Throughput Microbore LC-MS Lipidomics to Investigate APOE Phenotypes

Darshak Gadara[1], Vratislav Berka[1], Zdenek Spacil[1]*

[1] RECETOX Centre, Faculty of Science, Masaryk University, Brno, 625 00, Czech Republic

Chemicals and Reagents……………………………………………………………………………………2

Cerebral Organoid Culture………………………………………………………………………….……..2

Table S1: Characterized lipid species of single cerebral organoid with respective retention time, precursor and product ions, collision energy, and %CV of batch analysis…………………………………………………….……2

Table S2: Concentration (ng/mL) of lipid internal standards……………………………….................................8

Table S3: Gradient and flow rate optimization for the microbore column (1mm i.d). Four different flow rates. 50, 75, 100, and 150 µl/min. were acquired. each at three gradient lengths (~11, ~22 and ~33 min) with a 1.5 min re-equilibration……………………………………………………………………………………………….…9

Table S4: Peak height of lipid internal standards for microbore (1 mm i.d., 0.1ml/min flow rate) and narrow-bore separation (2.1 mm i.d., 0.1ml/min flow rate)…………………………………………………………………..…9

Table S5: Full width at half maxima (FWHM) of 16 lipid internal standards for microbore (1 mm i.d., 0.1ml/min flow rate) and narrow-bore separation (2.1 mm i.d., 0.1ml/min flow rate)……………………………………..10

Figure S1: Relative chromatographic separation for three phospholipid species PC 33:1, PC O-34:1 and PC P-34:0…………………………………………………………………………………………………...…..11

Figure S2: Relative chromatographic separation for four triglyceride species TG 52:0, TG 52:1, TG 52:2, and TG 52:3. ………………………………………………………………………………………………...………12

Figure S3: ESI MS/MS spectra of d7-cholesterol……………………………………………………….………13

Lipidomics Standards Initiative (LSI) Reporting Checklist…………………………………………………14

References…………………………………………………………………………………………………..…16

Chemicals and Reagents

Acetonitrile (cat. #00136878) was from Honeywell. Isopropanol (cat. #16267802) was from Biosolve. The ammonium format was from Sigma Aldrich. Ultrapure water was produced from a purification system (Simplicity 185. mfr. #SIMS600CP. Millipore Corp). Mass spec lipid standards SPLASH Lipidomics (cat. #330707) and Cer/Sph Mixture I (cat. #LM6002) were purchased from Avanti Polar Lipids (Alabaster, USA).

Cerebral Organoid Culture

Cell culture experiments are performed as per the published manuscript[1]. Two human iPSC lines used in this study were passaged and maintained using standard feeder-free culture protocols. In brief, feeder-free cultures were grown on Matrigel-coated plates (Corning) in mTeSRTM1 medium (STEMCELL Technologies) supplemented with half of the recommended dose of ZellShield® (Minerva Biolabs). Cells were passaged using 0.5 mM EDTA (Thermo Fisher Scientific) in PBS or manually. The "sAD-E4" cell line was derived from a patient with a sporadic form of AD with APOE4/4 status. The isogenic cell line "sAD-E3" was obtained by correction of APOE4/4 to APOE3/3. Both isogenic iPSC lines were kindly provided by Dr. Li-Huei Tsai and described previously by Lin and co-workers[2].

Cerebral organoids were generated using the protocol described previously[3,4]. Briefly, for spheroid formation. Cells were plated at day 0 into non-adherent V-shaped 96-well plates at 2000-3000 cells in 150 µl of mTeSRTM1 medium with 50 µM ROCK inhibitor (S1049. Selleckchem). Plates were centrifuged for 2 min at 200 g to facilitate spheroid formation. Non-adherent cell culture plates were prepared with poly(2-hydroxyethyl methacrylate) (poly-HEMA; P3932. Merck) coating; 3) On day 2. the cell culture medium was exchanged for fresh mTeSRTM1 without ROCK inhibitor. When spheroids reached the size of 400 - 600 µm, fresh Neural Induction Medium[4] was added every day for six days (usually from day 3 to day 8). Twelve organoids were transferred to one 6 cm cell culture dish the next day. The remaining medium was aspirated, and dry organoids were embedded in 7 µl of cold GeltrexTM (Thermo Fisher). GeltrexTM was left to solidify as hanging drops on the inverted cell culture dish for 10 min at 37 °C. Solidified GeltrexTM drops with organoids were gently detached from the bottom and cultured without shaking in Cerebral Organoid Differentiation Medium (CODM) without vitamin A for seven days. Subsequently, organoids were cultured in CODM with vitamin A and were moved on an orbital shaker on day 26 (± two days). CODM was changed three times a week by aspirating at least half of the old medium and replacing it with a fresh medium. ZellShield® (Minerva Biolabs) was used as the contamination preventive in all media.

Table S1: Characterized lipid species of single cerebral organoid with respective retention time, precursor and product ions, collision energy, and %CV of batch analysis

	Lipid Class	Lipid Species	Retention Time (Min)	Precursor Ion (m/z)	Product Ion (m/z)	Collision Energy (eV)	%CV
1	Acyl carnitines	CAR 20:0	4.86	456.4	85.1	25	4.2
2	Acyl carnitines	CAR 20:1	4.17	454.4	85.1	25	4.5
3	Acyl carnitines	CAR 22:0	5.61	484.4	85.1	25	4.4
4	Acyl carnitines	CAR 22:1	4.91	483.4	85.1	25	4.8
5	Acyl carnitines	CAR 24:1	5.63	511.5	85.1	25	4.2
6	Acyl carnitines	CAR 12:0	1.25	344.3	85.1	25	4.0
7	Acyl carnitines	CAR 14:0	1.74	372.3	85.1	25	4.8
8	Acyl carnitines	CAR 14:1	1.43	370.3	85.1	25	4.7
9	Acyl carnitines	CAR 14:2	1.22	368.3	85.1	25	7.6
10	Acyl carnitines	CAR 15:0	2.16	386.3	85.1	25	7.8
11	Acyl carnitines	CAR 16:0	2.71	400.4	85.1	25	4.3
12	Acyl carnitines	CAR 16:1	1.97	398.3	85.1	25	4.9
13	Acyl carnitines	CAR 18:0	4.03	428.4	85.1	25	4.7
14	Acyl carnitines	CAR 18:1	3.02	426.4	85.1	25	4.8

15	Chol. esters	CE 16:0	10.47	642.6	369.3	5	16.3
16	Chol. esters	CE 18:1	12.66	668.6	369.3	5	9.4
17	Chol. esters	CE 18:2	12.36	666.6	369.3	5	10.5
18	Chol. esters	CE 18:3	12.06	664.6	369.3	5	10.9
19	Chol. esters	CE 20:1	12.97	696.7	369.3	5	10.6
20	Chol. esters	CE 20:2	12.67	694.7	369.3	5	6.2
21	Chol. esters	CE 20:3	12.32	692.6	369.3	5	7.6
22	Chol. esters	CE 20:4	12.11	690.6	369.3	5	8.9
23	Chol. esters	CE 20:5	11.84	688.6	369.3	5	16.0
24	Chol. esters	CE 22:1	13.24	724.7	369.3	5	13.7
25	Chol. esters	CE 22:4	12.41	718.7	369.3	5	14.0
26	Chol. esters	CE 22:5	12.14	716.6	369.3	5	11.6
27	Chol. esters	CE 22:6	11.96	714.6	369.3	5	16.8
28	Chol. esters	CE 24:1	13.48	752.7	369.3	5	16.1
29	Chol. esters	CE 24:4	12.78	746.7	369.3	5	12.2
30	Free Cholesterol	FC	7.45	369.4	81.0	45	18.6
30	Free Cholesterol	FC	7.45	369.4	147.0	29	12.2
31	Ceramides	Cer 16:1;O2/16:0	7.48	510.6	236.3	33	19.5
32	Ceramides	Cer 16:1;O2/18:0	8.21	538.6	236.3	33	11.0
33	Ceramides	Cer 16:1;O2/22:0	9.54	594.6	236.3	33	9.4
34	Ceramides	Cer 16:1;O2/24:1	9.49	620.6	236.3	33	8.1
35	Ceramides	Cer 17:1;O2/16:0	7.85	524.6	250.3	33	17.0
36	Ceramides	Cer 17:1;O2/18:0	8.56	552.6	250.3	33	14.0
37	Ceramides	Cer 17:1;O2/24:0	10.39	636.6	250.3	33	8.5
38	Ceramides	Cer 17:1;O2/24:1	9.78	634.6	250.3	33	9.6
39	Ceramides	Cer 18:1;O2/14:0	7.48	510.5	264.3	33	19.0
40	Ceramides	Cer 18:1;O2/16:0	8.20	538.5	264.3	33	11.4
41	Ceramides	Cer 18:1;O2/18:0	8.90	566.6	264.3	33	14.1
42	Ceramides	Cer 18:1;O2/19:0	9.20	580.6	264.3	33	10.8
43	Ceramides	Cer 18:1;O2/20:0	9.51	594.6	264.3	33	8.2
44	Ceramides	Cer 18:1;O2/21:0	9.80	608.6	264.3	33	10.1
45	Ceramides	Cer 18:1;O2/22:0	10.10	622.6	264.3	33	6.2
46	Ceramides	Cer 18:1;O2/23:0	10.37	636.6	264.3	33	7.4
47	Ceramides	Cer 18:1;O2/24:0	10.62	650.6	264.3	33	7.0
48	Ceramides	Cer 18:1;O2/24:1	10.05	648.6	264.3	33	7.6
49	Ceramides	Cer 18:2;O2/16:0	7.59	536.5	262.3	33	17.1
50	Ceramides	Cer 18:2;O2/18:0	8.29	564.6	262.3	33	10.3
51	Ceramides	Cer 18:2;O2/20:0	8.97	592.6	262.3	33	18.1
52	Ceramides	Cer 18:2;O2/22:0	9.59	620.6	262.3	33	13.1
53	Ceramides	Cer 18:2;O2/24:1	9.56	646.6	262.3	33	10.2
54	Ceramides	Cer 19:1;O2/24:1	10.22	662.6	278.3	33	9.9
55	Ceramides	Cer 20:1;O2/24:0	11.08	678.6	292.3	33	8.9
56	Ceramides	Cer 20:1;O2/24:1	10.57	676.6	292.3	33	8.0
57	DG	DG 14:0_16:0	8.69	558.5	313.3	21	18.3
58	DG	DG 16:0_16:0	9.34	586.5	313.2	21	11.8
59	DG	DG 16:0_16:1	8.83	584.5	313.2	21	14.3
60	DG	DG 16:0_18:1	9.38	612.6	313.3	21	10.1
61	DG	DG 16:0_18:2	8.96	610.5	313.2	21	11.2
62	DG	DG 16:1_18:1	8.83	610.5	339.2	21	12.0
63	DG	DG 18:0_18:1	9.96	640.6	341.3	21	10.3
64	DG	DG 18:0_18:2	9.50	638.6	341.3	21	12.7
65	DG	DG 18:0_20:4	9.37	662.6	341.3	21	12.4
66	DG	DG 18:1_18:1	9.42	638.6	339.3	21	10.1
67	DG	DG 18:1_18:2	8.94	636.6	339.3	21	15.1
68	DG	DG 18:1_20:3	9.23	662.6	339.3	21	15.0
69	DG	DG 18:1_20:4	8.77	660.6	339.3	21	12.0
70	Dihydroceramide	Cer 18:0;O2/16:0	8.44	540.5	284.3	33	11.4
71	Dihydroceramide	Cer 18:0;O2/18:0	9.11	568.6	284.3	33	7.9
72	Dihydroceramide	Cer 18:0;O2/20:0	9.72	596.6	284.3	33	9.3
73	Dihydroceramide	Cer 18:0;O2/22:0	10.29	624.6	284.3	33	7.9

74	Dihydroceramide	Cer 18:0;O2/24:0	10.79	652.7	284.3	33	8.5
75	Dihydroceramide	Cer 18:0;O2/24:1	10.24	650.6	284.3	33	6.8
76	Dihydroceramide	Cer 18:1;O2/18:0	8.90	566.6	282.3	33	12.3
77	Dihydroceramide	Cer 18:1;O2/20:0	9.50	594.6	282.3	33	9.2
78	Dihydroceramide	Cer 18:1;O2/22:0	10.09	622.6	282.3	33	6.9
79	Dihydroceramide	Cer 18:1;O2/24:0	10.62	650.7	282.3	33	5.8
80	Dihydroceramide	Cer 18:1;O2/24:1	10.05	648.6	282.3	33	6.2
81	Hex2Cer	Hex2Cer 18:1;O2/16:0	7.32	862.6	264.3	41	15.6
82	Hex2Cer	Hex2Cer 18:1;O2/18:0	8.01	890.7	264.3	41	14.9
83	Hex2Cer	Hex2Cer 18:1;O2/22:0	9.29	946.7	264.3	41	15.1
84	Hex2Cer	Hex2Cer 18:1;O2/24:0	9.85	974.8	264.3	41	8.7
85	Hex2Cer	Hex2Cer 18:1;O2/24:1	9.22	972.7	264.3	41	10.6
86	Hex3Cer	Hex3Cer 18:1;O2/16:0	7.15	1024.7	264.3	41	19.0
87	Hex3Cer	Hex3Cer 18:1;O2/18:0	7.84	1052.7	264.3	41	25.6
88	Hex3Cer	Hex3Cer 18:1;O2/24:1	9.10	1134.8	264.3	41	19.1
89	HexCer	HexCer 18:1;O2/16:0	7.58	700.6	264.3	41	12.0
90	HexCer	HexCer 18:1;O2/18:0	8.28	728.6	264.3	41	8.7
91	HexCer	HexCer 18:1;O2/20:0	8.94	756.6	264.3	41	9.2
92	HexCer	HexCer 18:1;O2/22:0	9.54	784.7	264.3	41	7.0
93	HexCer	HexCer 18:1;O2/24:0	10.08	812.7	264.3	41	5.5
94	HexCer	HexCer 18:1;O2/24:1	9.49	810.7	264.3	41	7.4
95	LPC	LPC 26:0	6.38	636.5	184.1	29	11.4
96	LPC	LPC 28:0	7.08	664.5	184.1	29	13.6
97	LPC	LPC 14:0	1.79	468.3	184.1	29	4.7
98	LPC	LPC 16:0	2.81	496.3	184.1	29	3.1
99	LPC	LPC 16:1	2.06	494.3	184.1	29	4.0
100	LPC	LPC 18:0	4.12	524.4	184.1	29	3.5
101	LPC	LPC 18:1	3.12	522.4	184.1	29	3.6
102	LPC	LPC 18:2	2.27	520.3	184.1	29	31.8
103	LPC	LPC 20:1	4.26	550.4	184.1	29	3.1
104	LPC	LPC 20:4	2.21	544.3	184.1	29	8.7
105	LPC	LPC 20:5	1.71	542.3	184.1	29	14.5
106	LPC	LPC 22:1	5.00	578.4	184.1	29	5.0
107	LPC	LPC 22:4	3.15	572.4	184.1	29	13.0
108	LPC	LPC 22:6	2.12	568.3	184.1	29	7.0
109	LPC O	LPC O-16:0	3.32	482.4	184.1	29	4.4
110	LPC O	LPC O-18:0	4.48	510.4	184.1	29	4.0
111	LPC O	LPC O-18:1	3.59	508.4	184.1	29	4.0
112	LPC O	LPC P-16:0	3.23	480.3	184.1	29	3.8
113	LPC O	LPC P-18:0	4.41	508.3	184.1	29	3.5
114	LPC O	LPC P-18:1	3.49	506.3	184.1	29	3.7
115	LPE	LPE 16:0	2.89	454.3	313.3	25	10.8
116	LPE	LPE 18:0	4.22	482.3	341.3	25	6.2
117	LPE	LPE 18:1	3.20	480.3	339.3	25	27.1
118	PC	PC 28:0	6.63	678.5	184.1	37	14.6
119	PC	PC 30:0	7.35	706.5	184.1	37	12.8
120	PC	PC 30:1	6.78	704.5	184.1	37	19.1
121	PC	PC 30:2	6.50	702.5	184.1	37	13.2
122	PC	PC 31:0	7.69	720.5	184.1	37	12.3
123	PC	PC 31:1	7.13	718.5	184.1	37	11.3
124	PC	PC 32:0	8.04	734.6	184.1	37	15.2
125	PC	PC 32:1	7.42	732.5	184.1	37	17.8
126	PC	PC 32:2	6.95	730.5	184.1	37	102.9
127	PC	PC 32:3	6.63	728.5	184.1	37	14.8
128	PC	PC 33:1	7.80	746.6	184.1	37	12.5
129	PC	PC 33:2	7.23	744.6	184.1	37	11.0
130	PC	PC 34:0	8.69	762.6	184.1	37	14.4
131	PC	PC 34:1	8.10	760.6	184.1	37	12.1
132	PC	PC 34:2	7.58	758.6	184.1	37	15.5
133	PC	PC 34:3	7.27	756.5	184.1	37	44.8

134	PC	PC 34:4	6.75	754.5	184.1	37	12.4
135	PC	PC 35:1	8.42	774.6	184.1	37	12.2
136	PC	PC 35:2	7.83	772.6	184.1	37	13.7
137	PC	PC 36:1	8.75	788.6	184.1	37	11.2
138	PC	PC 36:2	8.17	786.6	184.1	37	12.4
139	PC	PC 36:3	7.66	784.6	184.1	37	40.7
140	PC	PC 36:4	7.46	782.6	184.1	37	16.6
141	PC	PC 36:5	6.99	780.5	184.1	37	24.4
142	PC	PC 37:4	8.74	796.6	184.1	37	25.8
143	PC	PC 38:1	9.30	816.6	184.1	37	13.4
144	PC	PC 38:2	8.77	814.6	184.1	37	15.7
145	PC	PC 38:3	8.30	812.6	184.1	37	38.4
146	PC	PC 38:4	7.93	810.6	184.1	37	19.2
147	PC	PC 38:5	7.50	808.6	184.1	37	23.8
148	PC	PC 38:6	7.23	806.6	184.1	37	12.2
149	PC	PC 38:7	6.67	804.5	184.1	37	23.3
150	PC	PC 40:1	9.83	844.7	184.1	37	13.3
151	PC	PC 40:2	9.33	842.7	184.1	37	17.8
152	PC	PC 40:3	9.05	840.6	184.1	37	12.1
153	PC	PC 40:4	8.57	838.6	184.1	37	11.7
154	PC	PC 40:5	7.95	836.6	184.1	37	22.3
155	PC	PC 40:7	7.29	832.6	184.1	37	12.7
156	PC O	PC O-28:0	7.04	664.5	184.1	37	15.2
157	PC O	PC O-30:0	7.78	692.6	184.1	37	12.6
158	PC O	PC O-30:1	7.11	690.5	184.1	37	23.5
159	PC O	PC O-32:0	8.46	720.6	184.1	37	12.3
160	PC O	PC O-32:1	7.86	718.6	184.1	37	16.0
161	PC O	PC O-32:2	7.28	716.6	184.1	37	38.7
162	PC O	PC O-33:2	8.02	736.6	184.1	37	15.6
163	PC O	PC O-34:0	9.11	748.6	184.1	37	10.8
164	PC O	PC O-34:1	8.51	746.6	184.1	37	12.5
165	PC O	PC O-34:2	7.90	744.6	184.1	37	11.0
166	PC O	PC O-36:1	9.12	774.6	184.1	37	12.2
167	PC O	PC O-36:2	8.43	772.6	184.1	37	13.7
168	PC O	PC O-36:4	7.89	768.6	184.1	37	18.8
169	PC O	PC O-36:6	7.29	764.5	184.1	37	18.3
170	PC O	PC O-38:1	9.68	802.7	184.1	37	11.8
171	PC O	PC O-38:2	9.22	800.6	184.1	37	12.8
172	PC O	PC O-38:3	9.06	798.6	184.1	37	23.2
173	PC O	PC O-38:4	8.73	796.6	184.1	37	42.6
174	PC O	PC O-38:5	8.23	794.6	184.1	37	22.1
175	PC O	PC O-38:6	7.79	792.6	184.1	37	13.8
176	PC O	PC O-40:5	8.28	822.6	184.1	37	51.4
177	PC P	PC P-30:0	7.67	690.4	184.1	37	13.8
178	PC P	PC P-32:0	8.36	718.5	184.1	37	11.0
179	PC P	PC P-32:1	7.85	716.6	184.1	37	13.7
180	PC P	PC P-34:0	9.01	746.6	184.1	37	12.5
181	PC P	PC P-34:1	8.42	744.6	184.1	37	11.0
182	PC P	PC P-34:2	7.88	742.5	184.1	37	16.1
183	PC P	PC P-36:2	8.43	770.6	184.1	37	13.0
184	PC P	PC P-36:4	7.77	766.5	184.1	37	12.0
185	PC P	PC P-40:4	8.84	822.6	184.1	37	33.0
186	PC P	PC P-40:5	8.27	820.6	184.1	37	23.8
187	PC P	PC P-40:6	7.82	818.6	184.1	37	28.1
188	PC P	PC P-35:2	7.27	756.6	184.1	37	35.8
189	PC P	PC P-36:3	7.90	768.5	184.1	37	23.1
190	PC P	PC P-38:5	7.79	792.6	184.1	37	13.8
191	PE	PE 32:0	8.23	692.5	551.5	25	21.5
192	PE	PE 32:1	7.61	690.5	549.5	25	19.6
193	PE	PE 34:1	8.28	718.5	577.5	25	18.1

194	PE	PE 34:2	7.67	716.5	575.5	25	16.7
195	PE	PE 34:3	7.45	714.5	573.5	25	28.0
196	PE	PE 35:1	8.61	732.5	591.5	25	33.5
197	PE	PE 35:2	8.00	730.5	589.5	25	31.0
198	PE	PE 36:1	8.94	746.6	605.6	25	19.2
199	PE	PE 36:2	8.32	744.5	603.5	25	20.1
200	PE	PE 36:3	7.85	742.5	601.5	25	19.9
201	PE	PE 36:4	7.64	740.5	599.5	25	23.2
202	PE	PE 36:5	7.17	738.5	597.5	25	19.8
203	PE	PE 38:2	8.96	772.6	631.6	25	33.1
204	PE	PE 38:3	8.58	770.6	629.6	25	22.2
205	PE	PE 38:4	8.32	768.5	627.5	25	18.0
206	PE	PE 38:6	7.23	764.5	623.5	25	23.2
207	PE	PE 40:3	9.23	798.6	657.6	25	18.9
208	PE	PE 40:4	8.79	796.6	655.6	25	32.4
209	PE	PE 40:5	8.32	794.6	653.6	25	31.9
210	PE	PE 40:6	7.70	792.5	651.5	25	61.5
211	PE	PE 40:7	7.52	790.5	649.5	25	26.3
212	PE O	PE O-34:2	8.63	702.5	561.5	25	19.4
213	PE O	PE O-36:2	9.23	730.6	589.6	25	24.5
214	PE O	PE O-36:3	8.63	728.5	587.5	25	24.3
215	PE O	PE O-36:5	7.95	724.5	583.5	25	20.6
216	PE O	PE O-36:6	7.50	722.5	581.5	25	27.5
217	PE O	PE O-38:2	9.79	758.6	617.6	25	45.0
218	PE O	PE O-38:3	9.03	756.6	615.6	25	28.9
219	PE O	PE O-38:4	8.95	754.6	613.6	25	25.5
220	PE O	PE O-38:6	7.98	750.5	609.5	25	19.9
221	PE O	PE O-38:7	8.94	748.5	607.5	25	18.5
222	PE O	PE O-40:4	9.52	782.6	641.6	25	23.5
223	PE O	PE O-40:6	8.47	778.6	637.6	25	19.4
224	PE O	PE O-40:7	8.01	776.5	635.5	25	26.6
225	PE O	PE O-40:8	7.82	774.5	633.5	25	24.1
226	PG	PG 32:1	6.64	738.5	549.5	21	11.2
227	PG	PG 34:1	7.27	766.5	577.5	21	13.9
228	PG	PG 34:2	6.75	764.5	575.5	21	11.2
229	PG	PG 36:2	7.35	792.5	603.5	21	12.8
230	PG	PG 36:3	6.94	790.5	601.5	21	12.9
231	PG	PG 36:4	6.72	788.5	599.5	21	15.4
232	PG	PG 38:5	6.76	814.5	625.5	21	17.1
233	PI	PI 32:0	7.06	828.5	551.5	17	12.8
234	PI	PI 32:1	6.49	826.5	549.5	17	15.5
235	PI	PI 34:0	7.71	856.5	579.5	17	23.0
236	PI	PI 34:1	7.11	854.5	577.5	17	14.4
237	PI	PI 34:2	6.63	852.5	575.5	17	15.6
238	PI	PI 36:1	7.81	882.6	605.6	17	17.0
239	PI	PI 36:2	7.19	880.5	603.5	17	19.9
240	PI	PI 36:3	6.84	878.5	601.5	17	22.7
241	PI	PI 36:4	6.69	876.5	599.5	17	13.0
242	PI	PI 38:3	7.65	906.6	629.6	17	16.8
243	PI	PI 38:4	7.19	904.5	627.5	17	18.3
244	PI	PI 38:5	6.59	902.5	625.5	17	12.1
245	PI	PI 38:6	6.17	900.5	623.5	17	38.1
246	PI	PI 40:4	7.63	932.6	655.6	17	17.8
247	PI	PI 40:5	7.21	930.6	653.6	17	53.5
248	PI	PI 40:7	6.44	926.5	649.5	17	13.8
249	PS	PS 34:1	7.41	762.5	577.5	25	16.4
250	PS	PS 34:2	6.84	760.5	575.5	25	26.6
251	PS	PS 36:0	7.94	792.6	607.6	25	15.7
252	PS	PS 36:1	7.93	790.5	605.5	25	17.8
253	PS	PS 36:2	7.41	788.5	603.5	25	20.6

254	PS	PS 38:1	8.61	818.6	633.6	25	23.9
255	PS	PS 38:2	7.95	816.6	631.6	25	37.0
256	PS	PS 38:3	7.68	814.5	629.5	25	26.3
257	PS	PS 38:4	7.40	812.5	627.5	25	24.7
258	PS	PS 40:4	7.91	840.6	655.6	25	18.1
259	PS	PS 40:5	7.41	838.5	653.5	25	35.4
260	PS	PS 40:6	7.21	836.5	651.5	25	17.7
261	PS	PS 40:7	6.67	834.5	649.5	25	15.0
262	PS	PS 42:6	7.64	864.6	679.6	25	30.0
263	SM	SM 31:1	6.19	661.5	184.0	25	21.2
264	SM	SM 32:0	6.83	677.6	184.0	25	19.5
265	SM	SM 32:1	6.58	675.5	184.0	25	12.3
266	SM	SM 32:2	5.94	673.5	184.0	25	32.5
267	SM	SM 33:1	6.96	689.6	184.0	25	24.7
268	SM	SM 34:0	7.59	705.6	184.0	25	9.1
269	SM	SM 34:1	7.31	703.6	184.0	25	11.3
270	SM	SM 34:2	6.69	701.6	184.0	25	14.4
271	SM	SM 35:1	7.83	717.6	184.0	25	11.0
272	SM	SM 35:2	7.53	715.6	184.0	25	17.2
273	SM	SM 36:1	7.98	731.6	184.0	25	14.8
274	SM	SM 36:2	6.62	729.6	184.0	25	10.6
275	SM	SM 37:1	8.40	745.6	184.0	25	9.4
276	SM	SM 37:2	7.87	743.5	184.0	25	10.7
277	SM	SM 38:1	8.72	759.6	184.0	25	13.1
278	SM	SM 38:2	7.25	757.6	184.0	25	31.7
279	SM	SM 38:3	6.75	755.6	184.0	25	12.4
280	SM	SM 39:1	9.03	773.7	184.0	25	14.0
281	SM	SM 40:0	8.75	789.7	184.0	25	10.2
282	SM	SM 40:1	9.35	787.7	184.0	25	9.4
283	SM	SM 40:2	7.64	785.7	184.0	25	40.9
284	SM	SM 40:3	7.44	783.6	184.0	25	19.0
285	SM	SM 41:0	9.03	803.7	184.0	25	37.1
286	SM	SM 41:1	9.62	801.7	184.0	25	11.1
287	SM	SM 41:2	9.03	799.7	184.0	25	28.0
288	SM	SM 42:1	9.92	815.7	184.0	25	12.4
289	SM	SM 42:2	9.31	813.7	184.0	25	9.2
290	SM	SM 43:2	9.59	827.7	184.0	25	31.7
291	SM	SM 44:2	9.87	841.6	184.0	25	10.9
292	SM	SM 44:3	8.60	839.6	184.0	25	11.3
293	SPB	SPB 18:0;O2	3.32	302.3	284.3	5	9.8
294	SPB	SPB 16:1;O2	1.90	272.3	254.3	5	16.1
295	SPB	SPB 18:1;O2	2.97	300.3	282.3	5	5.5
296	TG	TG 14:0_28:0	11.45	740.7	495.5	21	10.1
297	TG	TG 14:0_30:0	11.83	768.7	523.5	21	8.5
298	TG	TG 14:0_30:1	11.48	766.7	521.5	21	8.2
299	TG	TG 14:0_32:0	12.15	796.7	551.5	21	11.0
300	TG	TG 14:0_32:1	11.81	794.7	549.5	21	7.9
301	TG	TG 14:0_32:2	11.48	792.7	547.5	21	21.4
302	TG	TG 16:0_32:0	12.48	824.8	551.5	21	11.5
303	TG	TG 18:1_30:0	12.16	822.8	523.5	21	8.3
304	TG	TG 14:1_34:1	11.92	820.8	577.6	21	9.2
305	TG	TG 16:0_32:2	11.89	820.8	547.5	21	12.6
306	TG	TG 16:1_32:1	11.87	820.8	549.5	21	7.9
307	TG	TG 18:1_30:1	11.85	820.8	521.5	21	10.1
308	TG	TG 16:1_32:2	11.56	818.8	547.5	21	9.1
309	TG	TG 18:2_30:1	11.55	818.8	521.5	21	9.3
310	TG	TG 16:0_33:1	12.31	836.8	563.5	21	8.2
311	TG	TG 18:1_31:0	12.31	836.8	537.5	21	9.7
312	TG	TG 18:0_32:0	12.76	852.8	551.5	21	12.7
313	TG	TG 14:0_36:1	12.47	850.8	605.6	21	10.1

314	TG	TG 18:1_32:0	12.47	850.8	551.5	21	10.8
315	TG	TG 18:0_32:2	12.23	848.8	547.5	21	12.4
316	TG	TG 18:1_32:1	12.17	848.8	549.5	21	7.7
317	TG	TG 18:2_32:0	12.25	848.8	551.5	21	9.1
318	TG	TG 14:1_36:2	11.91	846.8	603.6	21	9.2
319	TG	TG 16:1_34:2	11.88	846.8	575.6	21	10.0
320	TG	TG 18:1_32:2	11.89	846.8	547.5	21	8.9
321	TG	TG 18:1_33:0	12.60	864.8	565.5	21	10.5
322	TG	TG 15:0_36:2	12.31	862.8	603.6	21	7.5
323	TG	TG 16:0_35:2	12.31	862.8	589.6	21	10.1
324	TG	TG 18:1_33:1	12.32	862.8	563.5	21	11.3
325	TG	TG 16:0_36:0	12.98	880.8	607.5	21	10.6
326	TG	TG 18:0_34:1	12.75	878.8	577.5	21	11.6
327	TG	TG 16:0_36:2	12.46	876.8	603.6	21	11.8
328	TG	TG 16:1_36:2	12.18	874.8	603.6	21	7.3
329	TG	TG 18:2_34:1	12.23	874.8	577.6	21	7.6
330	TG	TG 16:0_36:4	11.95	872.8	599.6	21	7.7
331	TG	TG 18:1_34:3	12.04	872.8	573.6	21	29.0
332	TG	TG 17:0_36:2	12.59	890.8	603.6	21	10.2
333	TG	TG 18:0_36:0	13.20	908.9	607.6	21	22.4
334	TG	TG 18:1_36:0	12.98	906.9	607.6	21	9.6
335	TG	TG 18:0_36:2	12.74	904.9	603.6	21	11.0
336	TG	TG 18:1_36:2	12.44	902.9	603.6	21	9.7
337	TG	TG 18:0_36:4	12.27	900.8	599.5	21	8.9
338	TG	TG 18:2_36:2	12.24	900.9	603.9	21	8.2
339	TG	TG 18:1_36:4	12.09	898.9	599.6	21	7.3
340	TG	TG 18:2_36:4	11.78	896.9	599.6	21	25.7
341	TG	TG 24:0_32:0	13.41	936.8	551.5	21	14.7
342	TG	TG 18:1_38:2	12.70	930.8	631.5	21	9.5
343	TG	TG 18:2_38:2	12.52	928.8	631.5	21	11.7
344	TG	TG 20:3_36:2	12.36	926.8	603.5	21	9.3
345	TG	TG 20:4_36:2	12.11	924.9	603.6	21	9.8
346	TG	TG 22:6_34:1	11.95	922.8	577.5	21	8.7
347	TG	TG 24:0_34:1	13.40	962.8	577.5	21	13.3
348	TG	TG 22:1_36:2	12.94	958.8	603.5	21	8.6
349	TG	TG 22:6_36:2	11.96	948.9	603.7	21	10.6
350	TG	TG 24:0_36:1	13.59	990.8	605.5	21	12.4
351	TG	TG 24:1_36:2	13.16	986.8	603.5	21	9.7

Table S2: Concentration (ng/mL) of lipid internal standards

**Lipid Standards**	**Conc. (ng/mL)**
15:0-18:1(d7) PC	640
15:0-18:1(d7) PE	20
15:0-18:1(d7) PG	120
17:0-14:1 PS	20
15:0-18:1(d7) PI	40
18:1(d7) Lyso PC	100
18:1(d7) Lyso PE	20
18:1(d7) Chol Ester	1400
15:0-18:1(d7) DAG	40
15:0-18:1(d7)-15:0 TAG	220
d18:1-18:1(d9) SM	120
C16 (d3) Carnitine	0.33
d18:1/12:0 Cer	25
d18:1/12:0 Lac Cer	25
d18:1/12:0 Gla Cer	25
(d7) Cholesterol	12500

Table S3: Gradient and flow rate optimization for the microbore column (1mm i.d). Four different flow rates. 50, 75, 100, and 150 µl/min. were acquired. each at three gradient lengths (~11, ~22 and ~33 min) with a 1.5 min re-equilibration.

Gradient - ~11 min		
Time (Min)	%A	%B
0.0	85	15
1.9	70	30
2.3	52	48
10.2	18	82
10.7	1	99
11.2	1	99

Gradient - ~22 min		
Time (Min)	%A	%B
0.0	85	15
3.7	70	30
4.7	52	48
20.5	18	82
21.4	1	99
22.3	1	99

Gradient - ~33 min		
Time (Min)	%A	%B
0.0	85	15
5.6	70	30
7.0	52	48
30.7	18	82
32.1	1	99
33.5	1	99

Table S4: Peak height of lipid internal standards for microbore (1 mm i.d., 0.1ml/min flow rate) and narrowbore separation (2.1 mm i.d., 0.1ml/min flow rate)

Peak Height		
	**Microbore**	**Narrowbore**
C16 (d3) Carnitine	22553	13333
15:0-18:1(d7) DG	28049	1733
15:0-18:1(d7)-15:0 TG	893480	273821
18:1(d7) Chol Ester	89502	13249
15:0-18:1(d7) PC	6021133	2391002
15:0-18:1(d7) PE	31724	7111
15:0-18:1(d7) PG	72020	18081
17:0-14:1 PS	7652	1479
15:0-18:1(d7) PI	12825	2814
18:1(d7) Lyso PC	557882	196313
18:1(d7) Lyso PE	3173	759
d18:1-18:1(d9) SM	791504	385759
d18:1/12:0 Cer	10220	2893

d18:1/12:0 Lac Cer	23839	6636
d18:1/12:0 Gla Cer	36561	8861
Cholesterol (d7)	12848	3999

Table S5: Full width at half maxima (FWHM) of 16 lipid internal standard for microbore (1 mm i.d., 0.1ml/min flow rate) and narrowbore separation (2.1 mm i.d., 0.1ml/min flow rate)

**Full width at half maxima (FWHM)**		
	**Microbore**	**Narrowbore**
C16 (d3) Carnitine	0.15	0.09
15:0-18:1(d7) DG	0.10	0.09
15:0-18:1(d7)-15:0 TG	0.09	0.09
18:1(d7) Chol Ester	0.08	0.08
15:0-18:1(d7) PC	0.11	0.10
15:0-18:1(d7) PE	0.10	0.10
15:0-18:1(d7) PG	0.09	0.09
17:0-14:1 PS	0.22	0.26
15:0-18:1(d7) PI	0.09	0.08
18:1(d7) Lyso PC	0.14	0.09
18:1(d7) Lyso PE	0.21	0.17
d18:1-18:1(d9) SM	0.09	0.10
d18:1/12:0 Cer	0.09	0.11
d18:1/12:0 Lac Cer	0.09	0.09
d18:1/12:0 Gla Cer	0.09	0.09
Cholesterol (d7)	0.09	0.08

Microbore (1 x 100 mm) separation at flow rate - 0.1 ml/mi

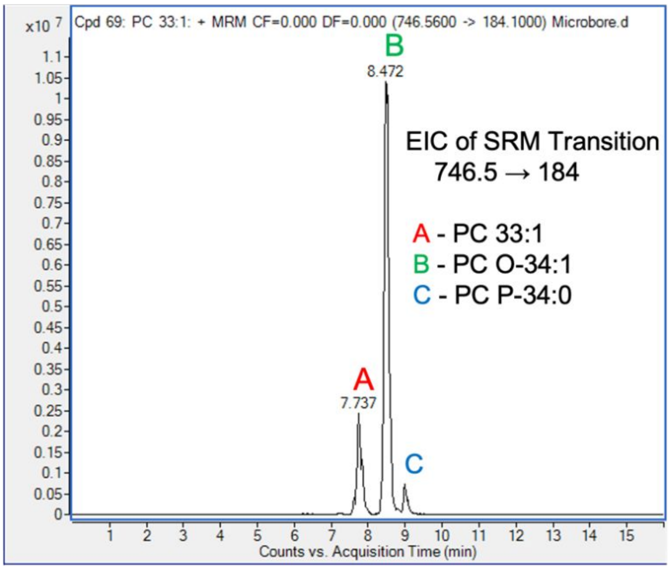

Narrowbore (2.1 x 100 mm) separation at flow rate - 0.42 ml/mi

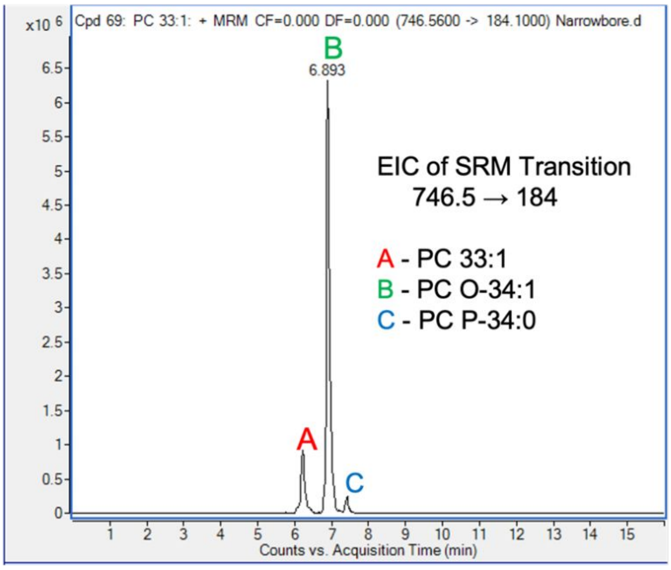

Figure S1: Relative chromatographic separation for three phospholipid species PC 33:1, PC O-34:1 and PC P-34:0

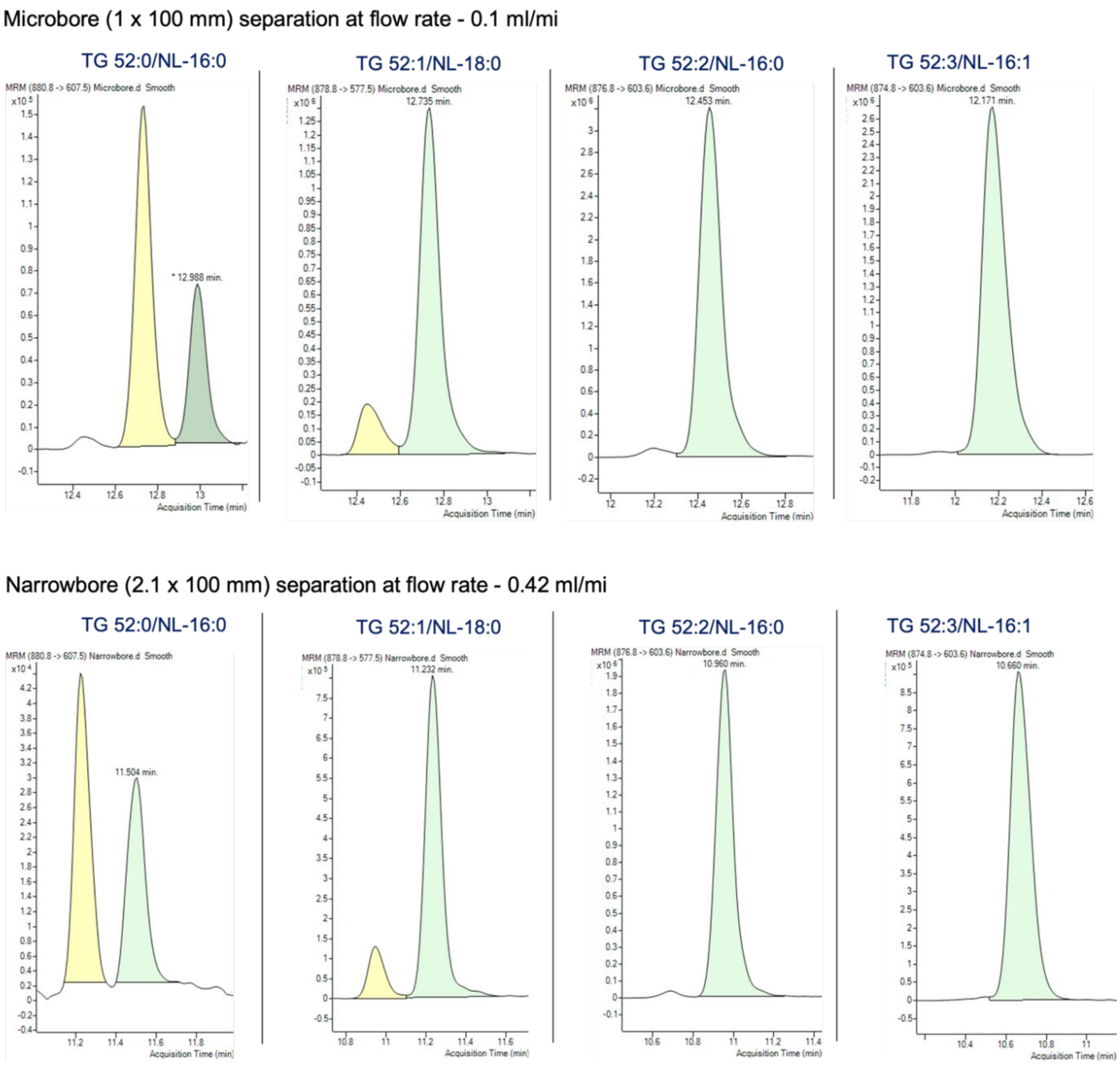

Figure S2: Relative chromatographic separation for four triglyceride species TG 52:0, TG 52:1, TG 52:2, and TG 52:3.

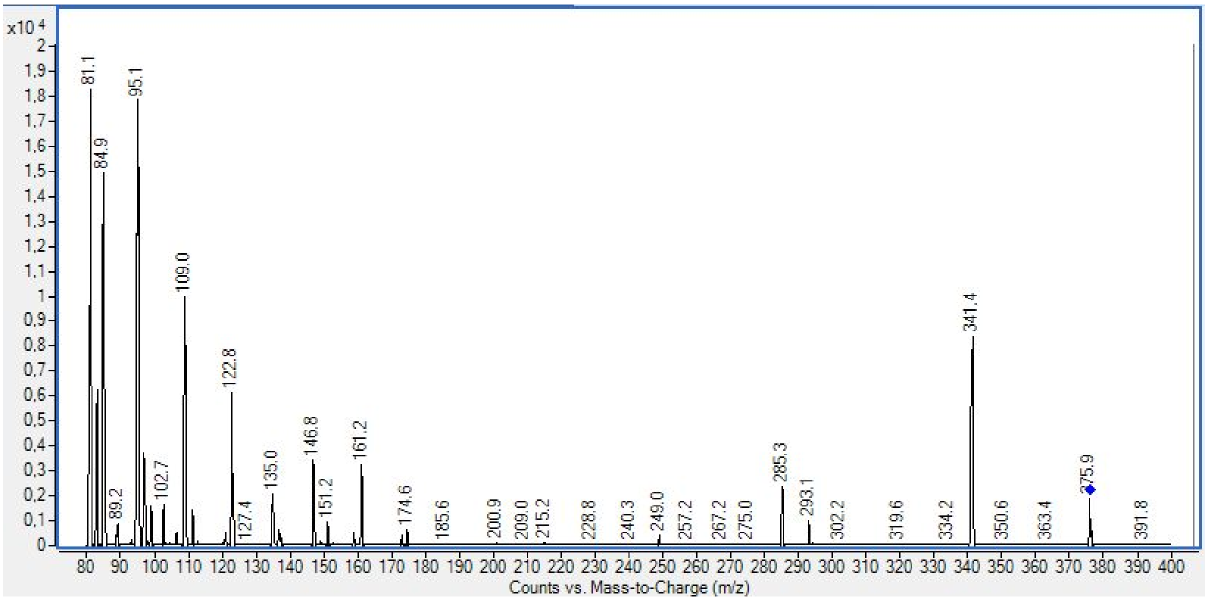

Figure S3: ESI MS/MS spectra of d7-cholesterol

Lipidomics Standards Initiative (LSI) Reporting Checklist

Separation Workflow

Created by https://lipidomicstandards.org, version v2.2.0

Overall study design

Title of the study	High-Throughput Microbore LC-MS Lipidomics to Investigate APOE Phenotypes		
Document creation date	09/06/2023	Corresponding Email	spacil@recetox.muni.cz
Principle investigator	Darshak Gadara	Is the workflow targeted or untargeted?	Targeted
Institution	Masaryk University	Clinical	No

Lipid extraction

Extraction method	1-phase system	1-phase system	Isopropanol
pH adjustment	None	Were internal standards added prior extraction?	No

Analytical platform

Number of separation dimensions	One dimension	MS vendor	Agilent
Separation Type 1	LC	Ion source	ESI
Separation Mode 1	NP	MS Level	MS1, MS2
Separation window (1) for lipid analyte selection (±) in minutes	0	Mass resolution for detected ion at MS1	Low resolution
RT verified by standard	Yes	Resolution at MS1	Unit
CCS verified by standard	No	Mass window for precursor ion isolation (in Da total isolation window)	0.7
Separation of isobaric/isomeric interferece confirmed	No	Mass resolution for detected ion at MS2	Low resolution
Model for separation prediction	No	Resolution at MS2	Unit
MS type	QQQ	Was/Were additional dimension/techniques used	No

Quality control

Blanks	Yes	Quality control	Yes
Type of Blanks	Extraction blank	Type of QC sample	Sample pool

Method qualification and validation

Method validation	No

Reporting

Are reported raw data uploaded into repository?	Available on request	Additional comments	-
Raw data upload	Available on request		

Sample Descriptions

Cerebral Organoid / Human / Cells

Provided information	-	Additives	None
Temperature handling original sample	4-8 °C	Were samples stored under inert gas?	No
Instant sample preparation	Yes	Additional preservation methods	No
Storage temperature	-80 °C	Biobank samples	No

Lipid Class Descriptions

1) PC[M+H]+ / Lipid identification

Lipid class	PC	Check isomer overlap	No
MS Level for identification	MS2	RT verified by standard	Yes
Identification level	Species level	Separation of isobaric/isomeric interferece confirmed	No
Polarity mode	Positive	Model for separation prediction	Yes
Type of positive (precursor)ion	[M+H]+	Additional dimension/techniques	-
Fragments for identification<br>Fragment name<br>HG(PC,184)		Lipid Identification Software	Targeted Lipid Profiling from Literature
Isotope correction at MS2	No	Data manipulation	-
MS2 verified by standard	No	Nomenclature for intact lipid molecule	Yes
Background check at MS2	Yes	Nomenclature for fragment ions	N/A
Did you presume assumptions for identification?	No	Further identification remarks	-

1) PC[M+H]+ / Lipid quantification

Quantitative	No	Batch correction	No
Normalization to reference	Yes	Further quantification remarks	-

REFERENCES

(1) Nemergut. M.; Marques. S.; Uhrik. L.; Vanova. T.; Nezvedova. M.; Gadara. D. C.; Jha. D.; Tulis. J.; Blechova. V.; Iglesias-Planas. J.; Kunka. A.; Legrand. A.; Hribkova. H.; Pospisilova. V.; Sedmik. J.; Raska. J.; Prokop. Z.; Damborsky. J.; Bohaciakova. D.; Spacil. Z.; Hernychova. L.; Bednar. D.; Marek. M. Domino-like Effect of C112R Mutation on APOE4 Aggregation and Its Suppression by Alzheimer's Disease Drug Candidate. *bioRxiv* **2022**. 2022.10.09.511473. https://doi.org/10.1101/2022.10.09.511473.

(2) Lin. Y. T.; Seo. J.; Gao. F.; Feldman. H. M.; Wen. H. L.; Penney. J.; Cam. H. P.; Gjoneska. E.; Raja. W. K.; Cheng. J.; Rueda. R.; Kritskiy. O.; Abdurrob. F.; Peng. Z.; Milo. B.; Yu. C. J.; Elmsaouri. S.; Dey. D.; Ko. T.; Yankner. B. A.; Tsai. L. H. APOE4 Causes Widespread Molecular and Cellular Alterations Associated with Alzheimer's Disease Phenotypes in Human IPSC-Derived Brain Cell Types. *Neuron* **2018**. *98* (6). 1141-1154.e7. https://doi.org/10.1016/j.neuron.2018.05.008.

(3) Camp. J. G.; Badsha. F.; Florio. M.; Kanton. S.; Gerber. T.; Wilsch-Bräuninger. M.; Lewitus. E.; Sykes. A.; Hevers. W.; Lancaster. M.; Knoblich. J. A.; Lachmann. R.; Pääbo. S.; Huttner. W. B.; Treutlein. B. Human Cerebral Organoids Recapitulate Gene Expression Programs of Fetal Neocortex Development. *Proc Natl Acad Sci U S A* **2015**. *112* (51). 15672–15677. https://doi.org/10.1073/PNAS.1520760112/SUPPL_FILE/PNAS.1520760112.SD04.XLSX.

(4) Lancaster. M. A.; Knoblich. J. A. Generation of Cerebral Organoids from Human Pluripotent Stem Cells. *Nat Protoc* **2014**. *9* (10). 2329–2340. https://doi.org/10.1038/nprot.2014.158.

(5) Huynh. K.; Barlow. C. K.; Jayawardana. K. S.; Weir. J. M.; Mellett. N. A.; Cinel. M.; Magliano. D. J.; Shaw. J. E.; Drew. B. G.; Meikle. P. J. High-Throughput Plasma Lipidomics: Detailed Mapping of the Associations with Cardiometabolic Risk Factors. *Cell Chem Biol* **2019**. *26* (1). 71-84.e4. https://doi.org/10.1016/j.chembiol.2018.10.008.

(6) Miranda. A. M.; Bravo. F. V.; Chan. R. B.; Sousa. N.; Di Paolo. G.; Oliveira. T. G. Differential Lipid Composition and Regulation along the Hippocampal Longitudinal Axis. *Translational Psychiatry 2019 9:1* **2019**. *9* (1). 1–12. https://doi.org/10.1038/s41398-019-0478-6.

(7) Xuan. Q.; Hu. C.; Yu. D.; Wang. L.; Zhou. Y.; Zhao. X.; Li. Q.; Hou. X.; Xu. G. Development of a High Coverage Pseudotargeted Lipidomics Method Based on Ultra-High Performance Liquid Chromatography-Mass Spectrometry. *Anal Chem* **2018**. *90* (12). 7608–7616. https://doi.org/10.1021/acs.analchem.8b01331.

(8) Liebisch. G.; Vizcaíno. J. A.; Köfeler. H.; Trötzmüller. M.; Griffiths. W. J.; Schmitz. G.; Spener. F.; Wakelam. M. J. O. Shorthand Notation for Lipid Structures Derived from Mass Spectrometry. *J Lipid Res* **2013**. *54* (6). 1523–1530. https://doi.org/10.1194/jlr.M033506.

(9) Vaňková. Z.; Peterka. O.; Chocholoušková. M.; Wolrab. D.; Jirásko. R.; Holčapek. M. Retention Dependences Support Highly Confident Identification of Lipid Species in Human Plasma by Reversed-Phase UHPLC/MS. *Analytical and Bioanalytical Chemistry 2021* **2021**. 1–13. https://doi.org/10.1007/S00216-021-03492-4.

(10) Gadara. D.; Coufalikova. K.; Bosak. J.; Smajs. D.; Spacil. Z. Systematic Feature Filtering in Exploratory Metabolomics: Application toward Biomarker Discovery. *Anal Chem* **2021**. *93* (26). 9103–9110. https://doi.org/10.1021/ACS.ANALCHEM.1C00816.
